# New Grain Formation Mechanisms during Powder Bed Fusion

**DOI:** 10.3390/ma14123324

**Published:** 2021-06-16

**Authors:** Alexander M. Rausch, Julian Pistor, Christoph Breuning, Matthias Markl, Carolin Körner

**Affiliations:** 1 Chair of Materials Science and Engineering for Metals, Friedrich-Alexander-Universität Erlangen-Nürnberg, Martensstr. 5, 91058 Erlangen, Germany; christoph.breuning@fau.de (C.B.); matthias.markl@fau.de (M.M.); carolin.koerner@fau.de (C.K.); 2 Joint Institute of Advanced Materials and Processes, Friedrich-Alexander-Universität Erlangen-Nürnberg, Dr.-Mack-Str. 81, 90762 Fürth, Germany; julian.pistor@fau.de

**Keywords:** selective electron beam melting, nucleation, single crystal, equiaxed, crystal growth, grain structure, stray grains, microstructure, solidification, numerical simulation

## Abstract

Tailoring the mechanical properties of parts by influencing the solidification conditions is a key topic of powder bed fusion. Depending on the application, single crystalline, columnar, or equiaxed microstructures are desirable. To produce single crystals or equiaxed microstructures, the control of nucleation is of outstanding importance. Either it should be avoided or provoked. There are also applications, such as turbine blades, where both microstructures at different locations are required. Here, we investigate nucleation at the melt-pool border during the remelting of CMSX-4^®^ samples built using powder bed fusion. We studied the difference between remelting as-built and homogenized microstructures. We identified two new mechanisms that led to grain formation at the beginning of solidification. Both mechanisms involved a change in the solidification microstructure from the former remelted and newly forming material. For the as-built samples, a discrepancy between the former and new dendrite arm spacing led to increased interdentritic undercooling at the beginning of solidification. For the heat-treated samples, the collapse of a planar front led to new grains. To identify these mechanisms, we conducted experimental and numerical investigations. The identification of such mechanisms during powder bed fusion is a fundamental prerequisite to controlling the solidification conditions to produce single crystalline and equiaxed microstructures.

## 1. Introduction

The production of parts with suitable mechanical properties is a prerequisite for manufacturing processes. Since the rise of additive manufacturing techniques, such as powder bed fusion (PBF), great efforts have been undertaken to understand and control mechanical properties based on process parameters [1,2,3,4]. PBF gives the opportunity to adjust the microstructure and, consequently, the mechanical properties in a broad range. Research demonstrated that, in addition to the usual columnar structures, single crystalline [5,6,7] and equiaxed microstructures [2,8,9,10,11] can be produced and also changed within one part [10]. Although different microstructures can be realized, the basic physical mechanisms are not fully clear. Therefore, the interactions between the process parameters and solidification behavior needs to be understood in detail.

For both single crystals (SX) and equiaxed microstructures, the nucleation of new grains is a key factor. In the first case, nucleation is avoided and, in the latter, it is provoked intentionally. New grains can form during PBF due to a conventional columnar-to equiaxed-transition (CET) [2,12,13,14,15,16,17]. If this transition happens right at the beginning of solidification, equiaxed microstructures can be achieved [17]. The CET mechanism is well understood. However, there is evidence that it can not explain all new grains observable during PBF. Helmer et al. [10] found that the usual CET criterion based on local temperature gradients and solidification velocities was violated.

A different nucleation mechanism was found where new grains formed primarily close to the melt-pool border [18,19,20,21,22,23]. The reasons for this grain formation are not well understood. Different mechanisms have been proposed. Liu et al. [23] claimed that high temperature gradients at the melt pool border led to increased nucleation. Drezet et al. [22] suggested that the constitutional fragmentation of dendrite arms is responsible. Rausch et al. [24] proposed that remelting segregated microstructures could lead to increased constitutional undercooling at the melt-pool border. In the welding of Al alloys, high temperature precipitates only present at the melt-pool border are thought to cause nucleation [25]. A general consensus is missing.

In the present work, we gain deeper insights regarding how remelting previous microstructures can influence new grain formation. This work is based on the mechanism proposed by Rausch et al. [24], in that remelting of segregated microstructures causes nucleation. According to this hypothesis, nucleation at the melt-pool border should vanish for homogenized samples. Therefore, single-melt lines with different parameters were produced on top of as-built as well as homogenized single crystalline CMSX-4^®^ samples built by Powder Bed Fusion with a Electron Beam (PBF-EB). The material was used as an example as it can be built as single crystalline and is completely homogenizable after being built. Based on the experimental results and supported by numerical simulation, we propose modifiednucleation mechanisms for segregated as well as homogenized microstructures.

## 2. Materials and Methods

### 2.1. Experiments

#### 2.1.1. Feedstock Atomization and Powder Properties

The prealloyed CMSX-4^®^ feedstock was provided by Cannon Muskegon Group (Muskegon, MI, USA) and subsequently atomized by TLS GmbH und Co. Spezialpulver KG (Bitterfeld-Wolfen, Germany) by means of the electrode-induction-melting-gas-atomization (EIGA) process with argon gas. The powder characterization revealed a suitable powder quality for PBF-EB with spherical particles within the target fraction of 45–105 μm. The chemical composition was measured by inductively coupled plasma optical emission spectrometry (ICP OES) and lies within the CMSX-4^®^ alloy specification (compare Table 1). For more details about powder characterization, the reader is referred to [5,26].

#### 2.1.2. SX Fabrication by EB-PBF

The build process was carried out in an Arcam A2 machine (Arcam EBM, Gothenburg, Sweden) operating under EBM control 5.2 with a standard 60 kV accelerating voltage. The controlled vacuum of 2 × 10^−3^ mbar He pressure establishes stable processing conditions and prevents surface oxidation. In addition, preheating as well as intermediate heating by raster scanning with a defocused electron beam is applied to achieve a necessary degree of sintering and a constant build temperature of 1030 ${}^{\circ }$C. Furthermore, a layer thickness of 50 m was applied.

The 15 × 15 × 25 mm${}^{3}$ SX samples were achieved using a snake-like hatch pattern with line order 1, 0.9 Jmm${}^{-1}$ line energy, and 150 m line offset. Contouring was not applied. Twelve cuboid samples were produced in one build job.

#### 2.1.3. Heat Treatment of SX Samples

The heat treatment of the chosen samples consisted of solution annealing and two step aging. The solution annealing was performed in a Carbolite RHF 1400 furnace (Carbolite Gero GmbH & Co. KG, Neuhausen, Germany) at 1310 ${}^{\circ }$C for 35 min in air with subsequent quenching in water. The two step aging was achieved in a Gero LHTM 250/300 (Carbolite Gero GmbH & Co. KG, Neuhausen, Germany) under a controlled Argon flow of 1 L h^−1^ after prior evacuation of the chamber to 1 × 10^−5^ mbar. For the first aging, a temperature of 1140 ${}^{\circ }$C for 2 h was used, whereas the second aging was performed at 870 ${}^{\circ }$C for 20 h. This procedure leads to a completely homogeneous microstructure with no residual dendritic segregation and a defined $\gamma /\gamma^{\prime }$ microstructure [5,27,28]. The heat treatment can be seen in detail in Table 2.

#### 2.1.4. Single Line Remelting on SX Substrate

The SX cuboid samples in heat treated and as-built conditions were cut in slices of 3-mm thickness using an ATM Brillant 220 (ATM Qness GmbH, Mammelzen, Germany). For the remelting experiments, a cross section 3 mm below the top surface was used, to guarantee the same degree of segregation, as the segregation is known to vary in dependency of the build height in the as-built condition due to the EB-PBF in situ heat treatment. The specimens were properly aligned in the build chamber. The remelting of the single lines was performed by using contour lines of 1-mm spacing, whereas each 15 × 15 mm cuboid was remelted by 14 single-melt lines in total. Within one sample, the beam power and velocity were constant for each single line. Intermediate heating after each specimen was performed to establish a constant preheating temperature of ∼1000 ${}^{\circ }$C. To compare the single lines in the as-built and heat-treated samples, a set of equal parameters were used. The parameters used can be found in Table 3.

#### 2.1.5. Offset Hatching on the SX Substrate

**Figure 1:**
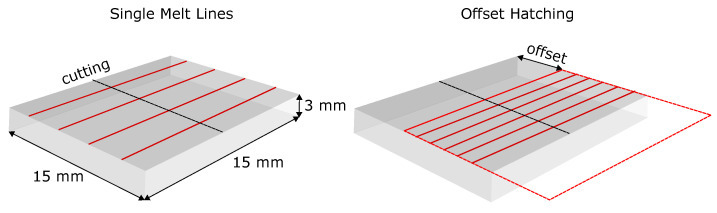
Schematic setup of the single-melt line and offset hatching remelting experiments. The offset was about 3.5 mm.

Additionally, a hatching remelting experiment in one slice was conducted. Therefore, the same hatching parameters were used as for the build up of the SX samples (see Section 2.1.2). The slice was aligned in the build chamber as described before. Then a snake-like hatching pattern was applied upon the specimen without powder. To obtain insights into the microstructure evolution at the beginning of hatching, an offset for the hatching pattern was applied (see Figure 1). Thus, it was possible to investigate the microstructure in the beginning without the influence of partially melted powder.

#### 2.1.6. Metallographic Preparation and Microstructural Investigation

The remelted samples were cut perpendicular to the melt lines and were metallographically prepared for the subsequent investigations in a standard manner by means of grinding with SiC and two step polishing with 3-m diamond particles and OPU suspension. V2A stain (a mixture of HCl, HNO_3_, H_2_O, and Vogels special reagent) were used to reveal the grain structure for the light microscopic investigations with a Leica DM6000 M (Leica Microsystems GmbH, Wetzlar, Germany). The grain structure and elemental segregation at the melt-pool border were investigated using scanning electron microscopy (SEM) and microprobe analysis performed on a Jeol JXA 8100 (JEOL Ltd., Tokyo, Japan) using non-etched samples. The grain orientation of the samples was investigated using electron backscatter diffraction (EBSD) with a FEI Helios NanoLab 600i FIB (FEI Company, Hillsboro, OR, USA).

Finally, Figure 1 shows, schematically, the single line and offset hatching experiments on SX slices.

### 2.2. Calculation of the Temperature Evolution

A semi-analytical heat conduction model was used to calculate the melt-pool evolution, temperature gradients, and cooling rates of the single-melt lines and hatching experiment. Despite its limitations and approximations, this model demonstrated its applicability for PBF processes [29,30,31]. The temperature *T* at time *t* were based on an analytical solution for the transient temperature response to a moving volumetric Gaussian heat source [32]:

(1) $$\begin{array}{cc} T\left(t,x,y,z\right)-T_{0}= & \frac{2\eta P}{c\rho {\left(\pi /3\right)}^{3/2}}\\ \; & \int_{0}^{t}\frac{1}{\sqrt{\phi_{x}\phi_{y}\phi_{z}}}exp\left(-\frac{3x{\left(t^{\prime }\right)}^{2}}{\phi_{x}}-\frac{3y{\left(t^{\prime }\right)}^{2}}{\phi_{y}}-\frac{3z{\left(t^{\prime }\right)}^{2}}{\phi_{z}}\right)dt^{\prime } \end{array}$$

where $T_{0}$ is defined as the preheating temperature, *P* is the beam power, η is the absorption coefficient, ρ the density, *c* the specific heat, and

(2) $$\phi_{i}=12\alpha \left(t-t^{\prime }\right)+\sigma_{i}^{2}\;for\;i=x,y,z$$

where the volumetric Gaussian beam shape (Equation (2)) is defined in each dimension by a beam width $\sigma_{i}$ and the thermal diffusivity α. The heat source motion is described by the coordinate system, where *x*, *y*, and *z* describe the distance from the point of interest to the transient location of the beam at time $t^{\prime }$. The piece-wise definition of the scan path prohibits the analytical integration of (Equation 1). Therefore, a Gaussian quadrature scheme, proposed by Stump et al. [33] was used to numerically integrate the temperature at a given time and location. The model neglects effects of fluid convection, latent heat release, radiation, and vaporization. The material properties are assumed to be constant and uniform. The estimated material parameters are summarized in Table 4.

## 3. Results

**Figure 2:**
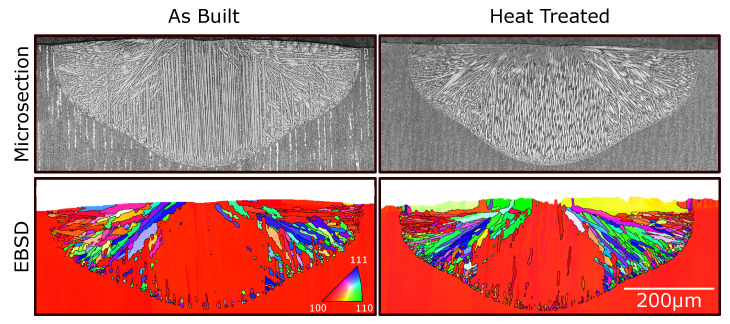
Microsections and EBSD images of a single-melt line (300 W and 0.25 m$s^{-1}$) in the as-built and heat treated SX base material.

Figure 2 shows microsections and EBSD images of representative single-melt lines (beam power 300 W and beam velocity 0.25 m s^−1^) for as-built and heat-treated SX base material (all other samples showed similar behavior). In all images, new grains appeared at the melt-pool border. A good agreement between different dendrite orientations in the microsections and new grains in their corresponding EBSD images was found.

For the as-built sample, clusters of small grains appear nearly equidistant perpendicular to the build direction of the SX. The mean distance between those clusters in the lower half of the melt pool lies around 17 μm. The primary dendrite arm spacing (PDAS) λ of the SX base material is around 13 μm. The PDAS in the melt line is roughly about 5 μm. In the heat treated sample, no periodicity of nucleation clusters is observable. Furthermore, the quantity of new grains is increased. In both cases, only grains towards the flanks of the melt pool survive and grow towards the center, whereas new grains close to the bottom are overgrown by the base material. The orientation of new grains appears to be random.

**Figure 3:**
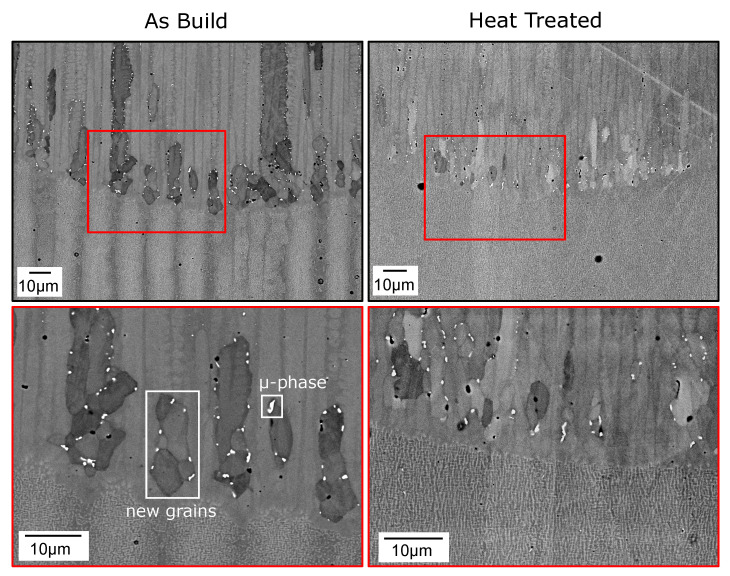
SEM images of the melt-pool bottom for the as-built and heat-treated samples (300 W and 0.25 m$s^{-1}$). The lower images show magnified areas marked in the upper ones. Two microstructural features are marked in the image bottom-left.

To gain further information about the location of new grains at the melt-pool border, Figure 3 depicts close-up SEM images. In addition to the contrast, new grains can be detected by white dots at their grain boundaries that are likely precipitates of the μ-phase [35]. In the as-built sample, new grains appear preferably at the border above the former interdentritic region of the SX material. This confirms a linkage between PDAS and the nucleation cluster spacing hinted in Figure 2. In the heat treated sample, new grains formed closer together. Additionally, nucleation took place after a specific distance of up to 5 μm from the melt-pool border.

**Figure 4:**
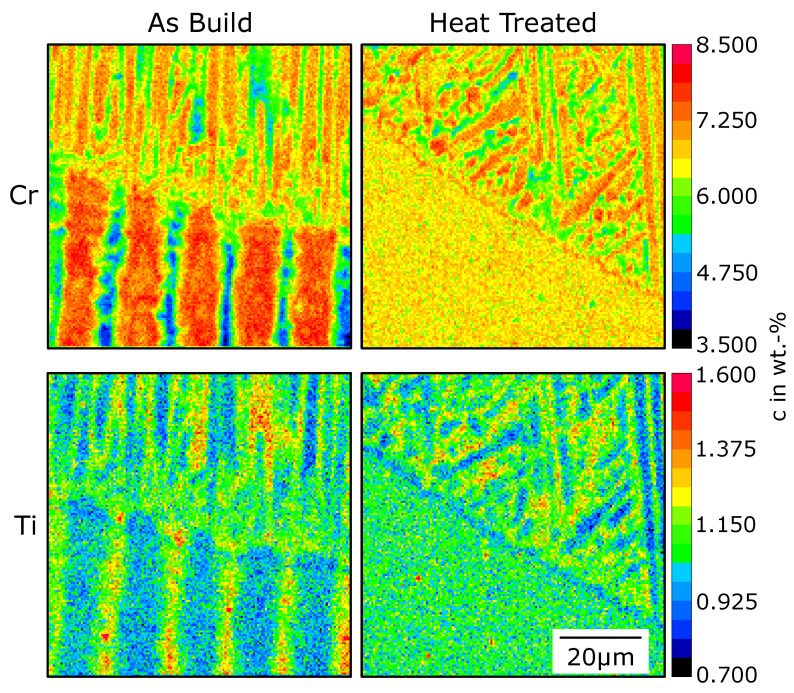
The concentrations of Cr and Ti for the as-built and heat-treated samples at the melt-pool border. The resolution of the concentration maps (0.5 μm) is additionally constrained by the micro probe measurement. For the given measurements, the effective resolution is in a range above 0.75 μm. Thus, the minimum and maximum values, especially for the fine microstructures in the melt pool, are lost due to averaging.

Figure 4 shows element concentration maps. An element with a partition coefficient k>1 (Cr) and k<1 (Ti) are depicted. The concentration of Cr is increased in the dendrite core and decreased in the interdentritic region. For Ti, it is vice versa. The two elements were chosen because of their good contrast in the micro-probe measurements. For the as-built sample, the coarse segregation profile from the SX at the bottom is clearly visible. In the melt line, the dendrite arm spacing is much smaller, leading to a shorter periodicity in the concentration variation. At the top, the dendrites grow parallel to the SX orientation. Close to the melt-pool border, the segregation profile is disturbed by new grains with different orientations.

For the heat treated sample, the SX is homogeneous. The finer dendrites in the melt pool are aligned with the SX towards the top right. However, along the melt-pool border, new grains in the segregation profile are apparent. Noticeable, for both elements, is a continuous increase (Cr) or depletion (Ti) at the melt-pool border separating the homogeneous SX and the dendritic segregation. This area is broader for Ti. For both elements, the thickness of this region is around 2 μm.

To further investigate the evolution of nucleation under changing solidification conditions, SX building parameters were used to melt a layer into a substrate build with the same SX parameters. This hatched layer was not aligned with the former hatching area of the substrate beneath (see Figure 1). Thus, the first melt lines during hatching can be examined without the influence of the powder.

**Figure 5:**
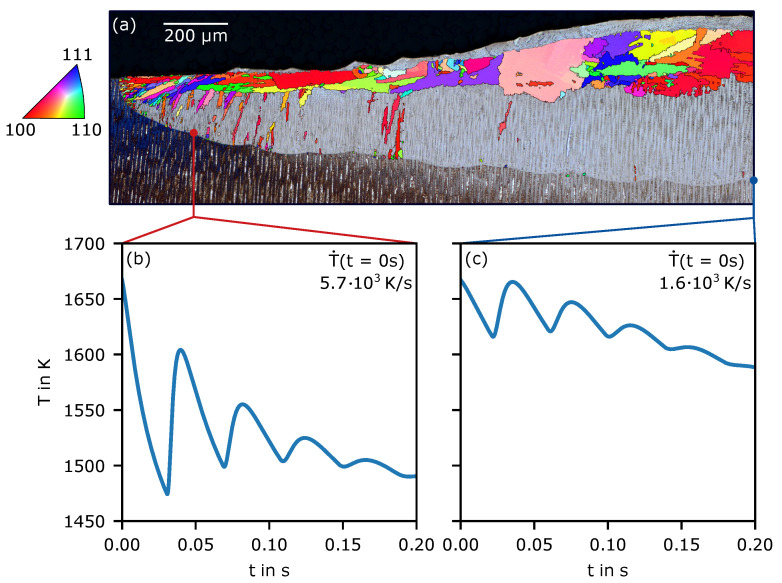
(**a**) Microsection with EBSD overlay for the hatched sample over the first 2 mm perpendicular to the beam movement. The EBSD signal of the SX substrate was removed. (**b**,**c**) show the temporal evolution of the temperature at the marked points in (**a**). (**b**) marks the point at the deepest melt-pool border where the first beam crosses the section. The same applies for (**c**) but at 2 mm. The time after the temperature falls below the liquidus temperature for the first time after melting is shown only. Additionally, the cooling rates $\dot{T}$ at the beginning of solidification for both locations are depicted.

Figure 5a shows the corresponding microesection and EBSD image over the first 2 mm perpendicular to the beam movement (lateral direction). Additionally, the temperature evolution at the deepest position melted was tracked at two positions (Figure 5b,c). The first was at the bottom of the first melt line and the second was after a 2-mm lateral distance. The time is depicted when solidification started. The temperature profile shows much greater variation in the beginning (Figure 5b) than after 2 mm (Figure 5c).

Due to the repeated passing of the beam, periodicity is apparent. As the beam moves further away with each additional melt line, the maximum temperature at a given point decreases with time. Generally, the temperature converges towards a steady state, where the base material temperature is increased. The temperature fluctuations are more pronounced in (Figure 5b) compared to (Figure 5c). This is because the lower base material temperature at the beginning. Equally, the cooling rate at the beginning at t=0 with 5700 K s^−1^ is about 3.6-times higher than in (Figure 5c) with 1600 K s^−1^.

The EBSD measurements show increased grain formation in the beginning at the melt-pool border (Figure 5a). Further hatching steadily decreased the amount of nuclei found at the border, until, after 2 mm, essentially no nucleation is apparent anymore. Only at the end of solidification, at the top of the sample, a classical CET transition is observable. Here, there will be no further investigation of this transition. In the microsection, it can be seen that material accumulated after several melt lines in the lateral direction (towards the right). The material transport is the topic of current research and will not be further addressed in this paper.

## 4. Discussion

For all considerations made in this section, a quasi binary system of Al in CMSX-4^®^ was used. Respectively, only the change in concentration of Al was considered. The rest was kept constant. Al was used because it can have the highest influence on the liquidus temperature based on its nominal concentration $c_{0}$, partition coefficient *k*, and liquidus slope *m* [36]. The corresponding material parameters are summarized in Table 5.

### 4.1. Grain Selection

From Figure 2, it is evident that, for both the as-built and heat-treated samples, new grains formed at the melt-pool border. As described in Rausch et al. [24], new grains survive only at the flanks, when their orientation with respect to the local temperature gradient is better than the SX orientation (growth competition [38]). At the bottom, the SX is always better aligned, and new grains are overgrown.

### 4.2. Nucleation in As-Built Samples

Several theories for melt-pool border nucleation exist. Liu et al. [23] claimed that high temperature gradients at the melt-pool border were responsible for new grains. However, no further explanation was given. Furthermore, constitutional fragmentation of dendrite arms were thought to produce new grains at the border [22]. However, due to the small dendrite arm spacing with poor evolution of secondary arms, we assume that such a mechanism is not relevant here.

For welding, equiaxed zones close to the melt-pool border are also known [25,39]. Gutierrez et al. [25] compared weld lines in pre-welded material and heat-treated samples. They demonstrated nucleation at the melt-pool border by welding in heat-treated samples. They proposed that high temperature precipitates formed during the solution heat treatment and acted as heterogeneous nucleation sites. These sites could only prevail during welding in a stagnant liquid layer close to the melt-pool border where the temperatures were the lowest in the melt.

In the bulk melt pool, they dissolved due to higher temperatures. By melting in pre-welded material, no equiaxed zones were found due to the absence of these precipitates. Up to now, there was no evidence for powder bed fusion regarding such an effect. Nevertheless, the presence of high melting impurities that already exist in the as-built state might lead to similar effects. However, if such a mechanism is always present at the melt-pool border it would not be possible to build SX. The proposed mechanism does not explain further preconditions, such as the local evolution of undercooling.

In a former study, we investigated the nucleation mechanism for as-built samples in single-melt lines in single crystalline Inconel 718 [24]. We found that the inhomogeneous concentration distribution from segregation was effectively homogenized by convection in the melt pool. However, at the melt-pool border, segregation leads to varying melt depths. In the deeper interdentritic regions, a concentration gradient was established, because convective flow is hindered in this confined space. Thus, a concentration gradient formed leading to constitutional undercooling at the start of solidification. This undercooling was believed to trigger nucleation at the melt-pool border. With lower cooling rates, the effect could vanish.

In Rausch et al. [24], only qualitative analysis was performed. Furthermore, the homogenization of a segregated microstructure due to remelting was only considered in 2D. Now, we derived a model giving more quantitative insights into the nucleation mechanism. Therefore, we look at the evolution of undercooling between regrowing remelted dendrites. This location has been identified in the as-built state as the most crucial for nucleation in Figure 3.

To determine the interdentritic undercooling at the start of solidification, the evolution of the concentration field between two dendrites must be known. The concentration above the melt-pool border is assumed to be at the nominal concentration $c_{0}$ (complete mixing of remelted segregations) when solidification starts as shown in Rausch et al. [24]. By remelting a presolidified dendritic microstructure, there is a discrepancy between the remelted dendrite arm spacing and the dendrites that will start to grow under different solidification conditions upon the remelted ones. For the single-melt lines (see Figure 2) or in the first melt lines of the hatching sample (see Figure 5), the new dendrite arm spacing is smaller (about 5 μm; compare with Figure 2).

According to Ridgeway et al. [40], there are two ways to reduce dendrite arm spacing during solidification. The first is due to secondary and tertiary arm growth. First, secondary arms grow into the interdentritic space. Then, tertiary arms can grow upon them and become primary arms. Second, nucleation caused by constitutional undercooling in the interdentritic region decreases the dendrite arm spacing. Our experiments show that the latter is the case.

To describe the concentration evolution, the Scheil equation [41,42] will be used. The basic approach to calculate the evolution in the melt during solidification is represented by

(3) $$c_{l}\left(1-k\right)df_{s}=\left(1-f_{s}\right)dc_{l}$$

with the concentration in the liquid $c_{l}$, the solid fraction $f_{s}$, and the partition coefficient *k*. Equation (3) can be solved numerically for $c_{l}$. Then, $c_{s}$ is obtained by $kc_{l}$. One issue of this equation is that it assumes a completely mixed and homogeneous concentration in the melt at all times. Thus, no constitutional undercooling can be expressed.

To solve this problem, the equation must be altered. Therefore, the elements in the melt can be redistributed. Normally, a concentration pile-up establishes ahead of the solidification front. The increased concentration at the solid–liquid interface steadily decreases towards the nominal concentration $c_{0}$ when moving away from the boundary. The size of this boundary layer can be expressed by the equivalent boundary layer $\delta_{c}$

(4) $$\delta_{c}=\frac{2D_{l}}{v_{i}}$$

with the liquid diffusion coefficient $D_{l}$ and the interdentritic solidification velocity $v_{i}$ [43]. By rearranging the concentration in the liquid, the increase per time step of the redistributed concentration field is

(5) $$dc_{l,D}=\frac{2c_{l}\left(1-f_{s}\right)}{f_{l,D}}$$

with the redistributed concentration at the interface $c_{l,D}$ and the part of the liquid fraction where the concentration is increased by diffusion $f_{l,D}$. The whole derivation of Equation (5) can be found in Appendix B.

The interdentritic solidification velocity $v_{i}$ can be calculated based on the KGT model [44]. A simplified correlation with the undercooling ΔT at the front was proposed by Gandin et al. [45]

(6) $$v_{i}=A\Delta T^{2}$$

with a constant prefactor *A*, which is 1 × 10^−4^ mK^−2^ s^−1^ in the original form. Köpf et al. [46,47] showed that this expression was capable of modeling the solidification velocity for nickel-base superalloys.

To determine the undercooling, simulation results are used. The temperature evolution at the deepest point in the middle of the melt pool is tracked over time when solidification starts (see Appendix C). The undercooling can then be determined by

(7) $$\Delta T=T_{l}+m\left(c_{l,D}-c_{0}\right)-T$$

with the liquidus slope *m* and the temporal evolution of temperature *T* at the melt pool bottom determined by simulation. By solving Equations (5)–(7) numerically over time, it is possible to obtain the evolution of concentration and undercooling between two dendrites. For Equation (6), the undercooling is calculated directly at the solid–liquid interface towards the interdentritic region based on the concentration at the interface (calculated with Equation (5)). We assumed that the temperature between two dendrites was approximately constant since the dendrite arm spacing was much smaller than the melt pool dimensions.

To obtain the spatial evolution of the concentration in the liquid, the dimension of a pile-up based on Equation (4) was used, and two pile-ups moving in opposite directions at a distance of λ at the current solid–liquid interfaces were superimposed. The material parameters to solve Equations (3)–(7) for the quasi binary system of Al in CMSX-4^®^ can be found in Table 5.

**Figure 6:**
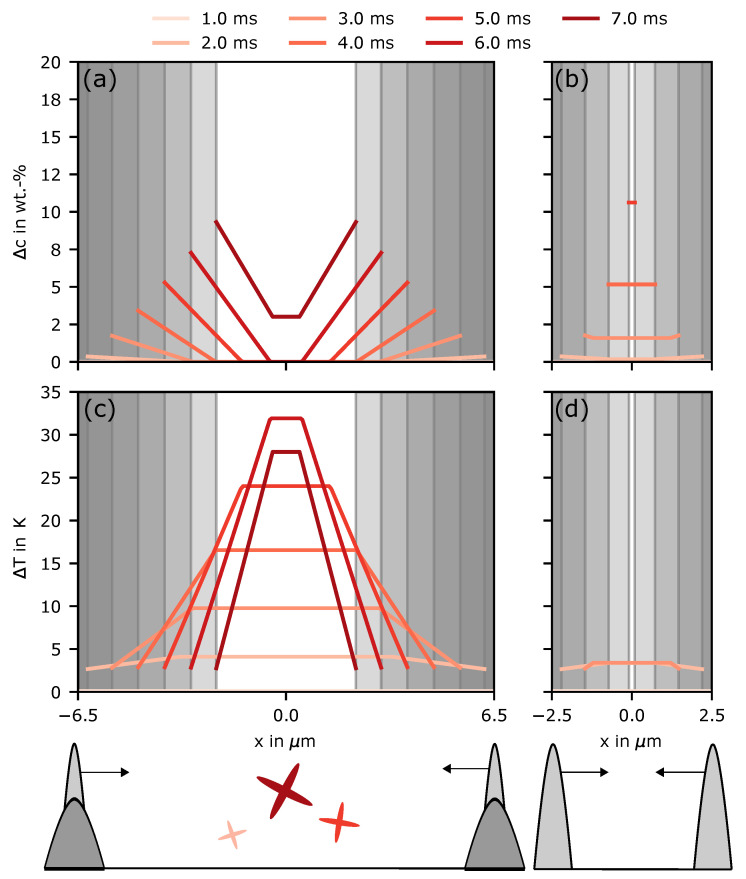
Concentration variation $\Delta c=c_{l}-c_{0}$ in the interdentritic space over several times for (**a**) coarse (λ=13 μm) and (**b**) fine (λ=5 μm) dendrites under the same solidification conditions obtained by simulation. (**c**,**d**) The corresponding undercooling ΔT evolution based on the local concentration/liquidus temperature and the local temperature evolution at the deepest melted point marked in Figure A2. The schematic at the bottom illustrates the condition of dendrites growing on a remelted structure with a bigger dendrite arm spacing (left) and dendrites growing with the equilibrium spacing for the same solidification conditions (right).

Based on Equations (3)–(7), Figure 6 shows the time dependent concentration distribution and undercooling between two dendrites with dendrite arm spacing λ of 13 μm (Figure 6a) as found in the experiments. Additionally, the same situation is depicted with a dendrite arm spacing of 5 μm (Figure 6b) as found in the single lines. This was done to compare the undercooling directly at the beginning when λ is equal to the remelted SX and if the base material has the equilibrium arm spacing as found in the experiments.

The maximum difference in the concentration Δc between the solid and the liquid was about 8 wt.-% at 6 ms for the SX spacing. Correspondingly, the maximum undercooling between the dendrites was over 30 K (Figure 6c). Before this value is reached in the melt, the initial transient must be passed where the pile-up is built up continuously as shown for the times before. For all times until 6 ms, the two evolving concentration profiles from both dendrites do not overlap due to the large spacing in the SX in relation to the equivalent boundary layer (compare Equation (4)).

For 7 ms, the profiles start to overlap. Then, the maximum undercooling decreases for further solidification. In Figure 6b, the situation for the equilibrium spacing is depicted. Here, the profiles overlap very fast. Therefore, the interdentritic concentration variation also increases very fast over 10 wt.-%, at 5 ms. Additionally, the concentration is nearly constant over the whole interdentritic space. According to the fast and homogeneous increase in concentration, the liquidus temperature shrinks fast as well. This leads to maximum undercooling below 5 K for a time of 3 ms (Figure 6d).

These simple considerations demonstrate two important facts: First, when new dendrites grow on remelted ones with larger spacing than the current solidification conditions, the undercooling in the interdentritic space can increase dramatically. This high undercooling can trigger new grain formation at the beginning of solidification. Second, when the large spacing is effectively reduced by either growth and/or nucleation and the equilibrium spacing is reached, the maximum undercooling is very low. Compared to the much higher undercooling for the large dendrite arm spacing, it is reasonable to assume that it is too low to provoke nucleation. This also fits the experimental observations, where only new grains can be found close to the melt-pool border.

For all estimations, we assumed that the temperature gradient was parallel with the remelted dendrite orientation. Up to now, the equations did not include an angular dependency. However, towards the flanks of the melt pool, the local temperature gradient was different from the SX orientation. Therefore, the following adjustment can be done. The angle between the temperature gradient and the dendrite orientation is α (see Figure A5). Thus, the effective distance between two dendrites increases when the dendrite and temperature gradient directions deviate. Thus, an effective dendrite arm spacing can be calculated and used in the aformentioned equations:

(8) $$\lambda_{\alpha }=\frac{\lambda }{\cos(\alpha )}$$

**Figure 7:**
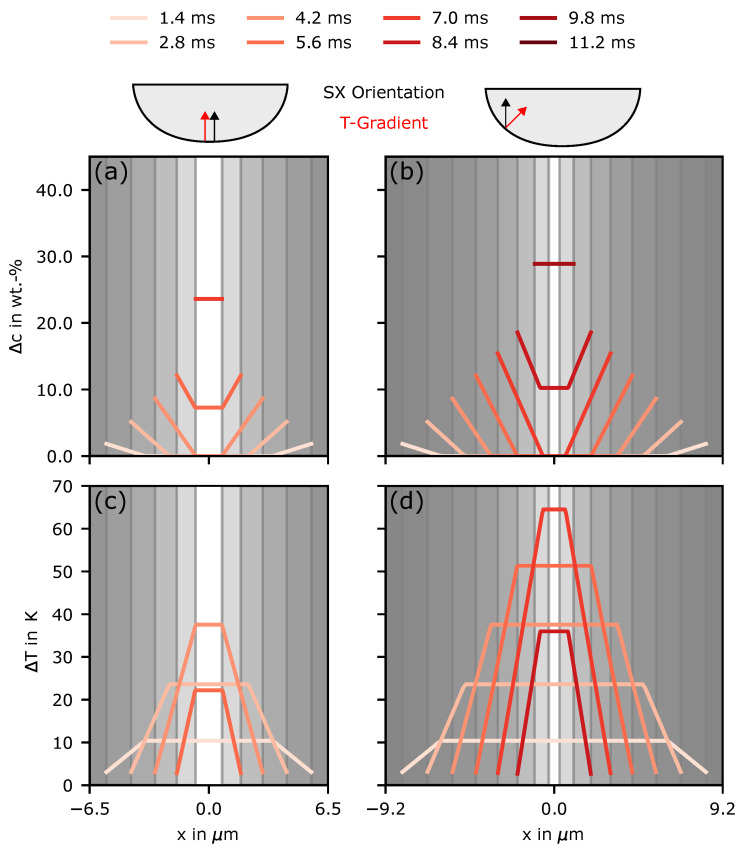
(**a**) Concentration variation $\Delta c=c_{l}-c_{0}$ in the interdentritic space over several times at the bottom of the first melt line (**a**) where the temperature gradient is parallel to the main growth direction of the underlying SX and at an angle of 45° towards the SX orientation (**b**). For (**a**,**b**) the same temperature evolution as in Figure 6 was used for better comparison. For (**b**), the effective dendrite arm spacing $\lambda_{\alpha }$ was calculated and used instead of the original λ of 13 μm. (**c**,**d**) The corresponding undercooling ΔT evolution based on the local concentration/liquidus temperature and the local temperature evolution at the point marked in Figure A2.

In the experiments (see Figure 2), it can be seen that at an angle of 45 towards the SX orientation, nucleation becomes even denser than at the bottom. This can also be triggered by an increased undercooling caused by a larger effective spacing. To show this effect, an angle of 45° was assumed, and the corresponding effective spacing $\lambda_{\alpha }$ was calculated. Figure 7 compares the results from Figure 6a,c with a situation of increased effective arm spacing. For better comparison, the same temperature evolution as for the bottom was considered for the increased spacing.

The results show that, for an increased effective arm spacing, the concentration fields overlap later at 10 ms (compare Figure 7b) instead of 6 ms (compare Figure 7a). Additionally, it takes longer to close the interdentritic space. Overall, both effects lead to an increased undercooling above 60 K. Thus, by considering a deviation of the grain and temperature gradient orientation, the maximum undercooling was roughly increased by a factor of 1.5. This can explain the increased nucleation towards the melt-pool flanks.

The effect of increased effective arm spacing may also lead to the situation where dendrites grow with the same arm spacing as found in the remelted structure, but nucleation is possible due to the increased effective arm spacing. This also correlates with the assumption that high deviations of the temperature gradient towards the build direction lead to nucleation as proposed by Helmer et al. [10].

**Figure 8:**
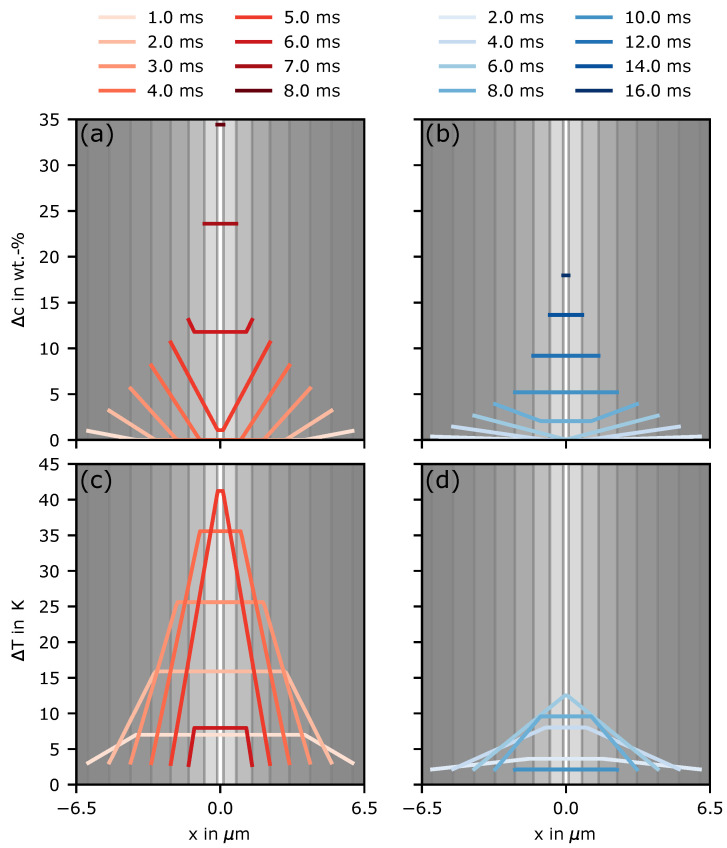
Concentration variation $\Delta c=c_{l}-c_{0}$ in the interdentritic space over several times in the first melt line (**a**) and after a distance of 2 mm during hatching (**b**). (**c**,**d**) The corresponding undercooling ΔT evolution based on the local concentration/liquidus temperature and the local temperature evolution at the deepest melted points marked in Figure 5.

To further test the predictions of the model, the conditions during hatching were also considered. Therefore, the solidification conditions at the bottom of the first melt line were compared to the conditions after 2 mm (see Figure 5). The corresponding temperature evolution at these positions was used from Figure 5b,c. The results are shown in Figure 8.

The maximum undercooling reached in the first melt line was over 40 K (Figure 8c). Based on the values found in Figure 6, nucleation can be expected. Due to the higher solidification velocities in the beginning, the concentration fields overlap first after 5 ms. Later, during hatching after a 2-mm lateral distance, the temperature evolution is not as steep as it is in the beginning, and the cooling rates are lower (compare Figure 5b,c). Accordingly, the solidification velocity shrinks.

As can be seen in Figure 8b, the concentration fields overlap after 6 ms, decreasing the potential constitutional undercooling. The lower cooling rates lead to a decrease in the thermal undercooling. In the end, the maximum undercooling achieved is below 15 K (Figure 8d). Thus, after 2 mm, less nucleation can be expected. Compared to the experimental results in Figure 5a, it is apparent that the model predicts the increased nucleation probability at the beginning and the essentially no nucleation after 2 mm correctly.

### 4.3. Nucleation in Heat-Treated Samples

For the heat-treated samples, the aforementioned theory based on a misfit in dendrite arm spacing can not explain the nucleation at the melt-pool border. First, we start with a discussion about the continuous concentration variation found in Figure 4 at the melt-pool border of heat-treated samples. There are two possible approaches to explain this phenomenon. Such a concentration layer could be produced either during melting or solidification. As shown in Figure 6, solute diffusion from the solid in the liquid leads to an increase in concentration close to the solidification front.

However, during melting, the opposite effect is apparent. Instead of an increase of the solute concentration in the liquid, a depletion region establishes in the solid [48]. This depletion zone, as seen for Ti, may explain the continuous concentration variation. However, diffusion in the solid is limited. The time where the solid–liquid interface stays at the turning point from melting to solidification is in the order of t=1 ms. Additionally, the diffusion coefficient at the liquidus temperature in the solid is in the range of $D_{s}=1\times 10^{-12}$ m^2^ s^−1^ [36]. Consequently, the effective diffusion length $l=\sqrt{4Dt}$ is only 63 nm. This can not explain the 2-m-thick layer found in Figure 4.

Thus, the concentration layer is formed during solidification. For cellular/dendritic growth, constitutional undercooling is required. When solidification starts, the concentration pile-up needs to built up first (initial transient) before constitutional undercooling is present. Until then, planar growth can be expected. This could explain the continuous concentration layer. The length of the initial transient can be estimated by [49,50]

(9) $$\frac{c_{s}}{c_{0}}=1-\left(1-k\right)\exp\left(-\frac{kvx}{D_{l}}\right)$$

with the concentration in the solid $c_{s}$ and the solidification distance *x*. Solving Equation (9) for the solidification distance *x* where $\frac{c_{s}}{c_{0}}$ is 0.99 (approximately steady state), the length of the initial transient is

(10) $$x_{T}=-D_{l}\frac{\ln\left(\frac{1-0.99}{1-k}\right)}{kv}$$

**Figure A3:**
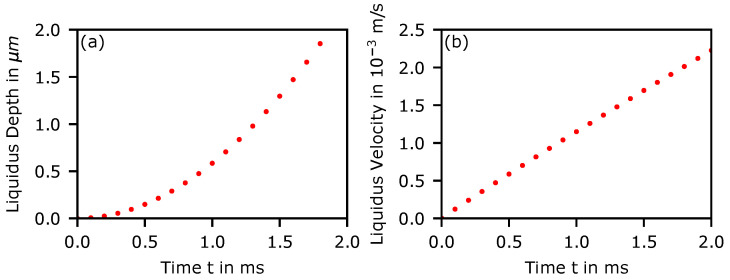
The time from the turning point over the (**a**) liquidus position and (**b**) liquidus velocity found in numerical simulation along the solidification pathway defined in Figure A2.

For the mean velocity found in the first 2 μm of about $2\times 10^{-3}$ m s^−1^ (see Figure A3), the length of the initial transient $x_{T}$ is 1.9 μm. This is in good agreement with the thickness of the concentration layer of about 2 μm as found in experiments.

Since a steady state is established, the constitutional undercooling ahead of the front increases. The evolution of the liquidus temperature $T_{l}$ ahead of the front can be calculated with

(11) $$T_{l}=T_{0}-m\Delta c\left(x_{F},t\right)$$

with the liquidus temperature $T_{0}$ at $c_{0}$ and the distance to the moving front $x_{F}$. To calculate the concentration pile-up, the time dependent solution from Tiller et al. [49] is used that also considers the initial transient region.

(12) $$\Delta c=c_{l}-c_{0}=\frac{\left(1-k\right)c_{0}}{k}\left(1-\exp\left(-\frac{kvx}{D_{l}}\right)\right)\exp\left(-\frac{vx_{F}}{D_{l}}\right)$$

**Figure A4:**
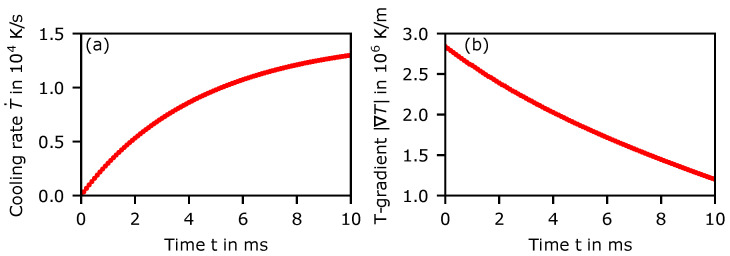
The time from the turning point over the (**a**) cooling rate and (**b**) temperature gradient found in numerical simulation along the solidification pathway defined in Figure A2.

**Figure A5:**
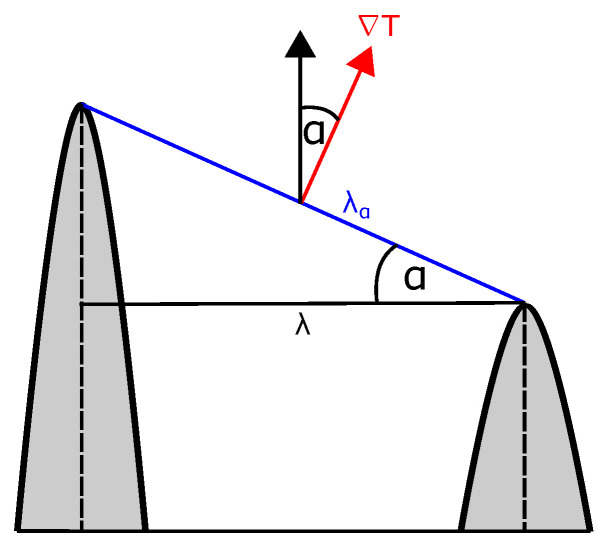
Effective dendrite arm spacing $\lambda_{\alpha }$ based on the orientation of the dendrite and local temperature gradient ∇T.

*x* is the already moved distance of the solidification front, and *v* is the solidification velocity (see Figure A4).

Based on Tiller et al. [49], the temperature ahead of the front is

(13) $$T=T_{0}-m\frac{c_{0}}{k}+\left|\nabla T\right|x_{F}$$

with the temperature gradient |∇T|. We assumed that the temperature at the solid–liquid interface is equal the local liquidus temperature. Thus, the difference between Equations (11) and (13) depicts the constitutional undercooling $\Delta T_{c}$ ahead of the front at a time *t* after the turning point (melting to solidification):

(14) $$\Delta T_{c}=T_{l}-T=m\left(\frac{c_{0}}{k}-\Delta c\left(x_{F},t\right)\right)-\left|\nabla T\right|x_{F}$$

From simulation, the temperature gradient, solidification velocity and front position are known over time (compare Figure A3 and Figure A4). Thus, the time-dependent evolution of the constitutional undercooling can be calculated.

**Figure 9:**
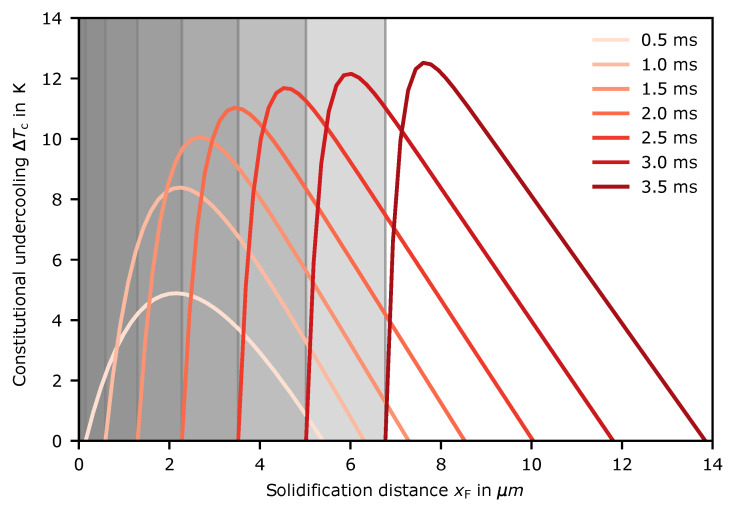
Constitutional undercooling $\Delta T_{c}$ over several times ahead of the solidification front.

The results are shown in Figure 9 for several times.

As can be seen, the constitutional undercooling increases over time. The maximum converges towards a saturation limit that is not reached during the considered times. After 5 μm, the undercooling is around 12 K. The experiments show that, at this point, the undercooling becomes high enough to destabilize the planar front. This is accomplished by new grain formation along the whole melt-pool border. After the planar front is decomposed, dendrite growth continues, and the constitutional undercooling ahead of the original planar front is decreased. After the transition, no further nucleation is observable.

## 5. Summary and Conclusions

In summary, we examined new grain formation in single-melt lines and offset hatching produced on top of single crystalline CMSX-4^®^ samples built by PBF-EB. The single lines were melted into as-built as well as heat-treated samples to investigate the influence of remelting segregated microstructures on nucleation. In both cases, new grains formed along the whole melt-pool border.

In the as-built case, nucleation occurred only when the current solidification conditions at the melt-pool border were different to the conditions that were present when the base material solidified. The most important and directly measurable quantity was the primary dendrite arm spacing. If the current and the previous spacings were similar, nucleation at the melt-pool border was suppressed, and SX manufacturing was possible. In all other cases, nucleation may occur due to increased interdentritic undercooling. Regarding the homogenized samples, increasing the constitutional undercooling at the beginning led to decomposition of a planar front and subsequently to new grain formation close to the melt-pool border.

The results and the proposed nucleation model can help to understand how new grain formation at the melt-pool border can be avoided to build single crystals and how it can be intentionally provoked to produce equiaxed microstructures by influencing the solidification conditions. 

## Figures and Tables

**Table 1:** Chemical composition of the CMSX-4^®^ powder material.

Element	Al	Co	Cr	Hf	Mo	Re	Ta	Ti	W	Ni
wt.-%	6.15	9.93	6.60	0.085	0.65	2.91	6.57	1.08	6.44	Bal.

**Table 2:** The heat treatment procedure for the single crystals (SX) samples.

Heat Treatment	Ramp 1	Ramp 2	Target	Cooling
Solution	RT–1000 ${}^{\circ }$C	1000–1310 ${}^{\circ }$C	1310 ${}^{\circ }$C	water
Annealing	20 K/min	5 K/min	35 min	
Ageing	RT–1000 ${}^{\circ }$C	1000–1140 ${}^{\circ }$C	1140 ${}^{\circ }$C	Argon
Annealing 1	20 K/min	5 K/min	2 h	
Ageing	RT–870 ${}^{\circ }$C	-	870 ${}^{\circ }$C	Argon
Annealing 2	5 K/min	-	20 h	

**Table 3:** The beam parameters for the single-line experiments. The line-energy stepping was 200 J m^‒1^.

Samples	Line Energy (P/v)	Beam Power *P*	Scan Speed *v*
1–4	800–1400 J m^‒1^	200–350 W	0.25 m s^‒1^
6–9	600–1400 J m^‒1^	300–700 W	0.50 m s^‒1^

**Table 4:** The material properties for CMSX-4^®^ used in simulation.

Property	Value	Reference
Thermal diffusivity (m^2^ s^−1^)	$3.2\times 10^{-6}$	[34]
Density (kg m^−3^)	8193	[34]
Specific heat (J kg^−1^ K^−1^)	925	[34]
Absorption coefficient	0.85	
Preheat temperature (${}^{\circ }$C)	1000	-
Liquidus temperature (${}^{\circ }$C)	1394	[28]

**Table 5:** Material parameters for a quasi binary system of Al and CMSX-4^®^.

Parameter	Value	Reference
Nominal concentration $c_{0}$ (wt.-%)	5.7	Table 1
Partition coefficient *k*	0.6	[37]
Diffusion coefficient (liquid) $D_{l}$ (m^2^ s^−1^)	$1\times 10^{-9}$	Appendix A
Diffusion coefficient (solid) $D_{s}$ (m^2^ s^−1^)	$1\;\times \;10^{-12}$	[36]
Liquidus slope *m* (K wt.-%^−1^)	−4	[37]

**Figure A1:**
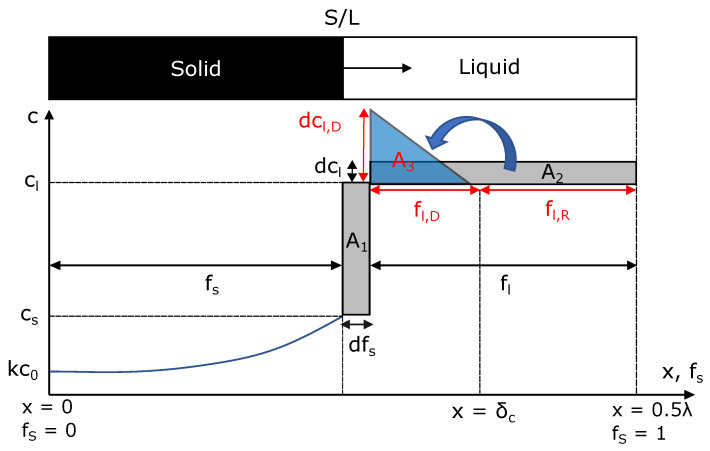
Mathematical approach of calculating the concentration distribution during solidification in the solid and liquid for the classical Scheil approach (areas $A_{1}$ and $A_{2}$) are considered as well as a new advanced approach for calculating a non-homogeneous distribution in the melt (additionally $A_{3}$ is considered). The concentration *c* is depicted in the solid ($c_{s}$) and the liquid ($c_{l}$) over the solid fraction $f_{s}$ at an arbitrary time during solidification. Equivalently to the solid fraction, the solidification distance *x* can be considered. The liquid fraction $f_{l}$ in the classical case and the split of this fraction into $f_{l,D}$ and $f_{l,R}$ for the advanced approach are shown.

**Figure A2:**
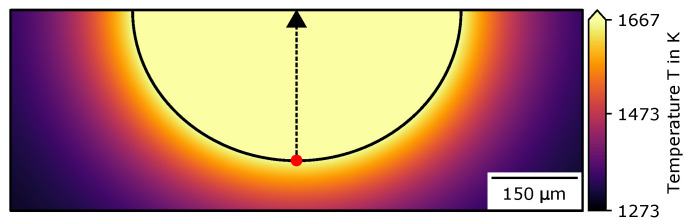
Snapshot of temperature field and melt pool geometry at the turning point perpendicular to the beam movement. The dashed line shows where the solidification pathway was examined. The red dot shows the point where the temperature evolution was tracked over time. The beam velocity and beam power for melting were 0.25 m s^−1^ and 300 W.

## Data Availability

The data presented in this study are available on request from the corresponding author.

## Appendix A. Liquid Diffusion Coefficient

The magnitude of the diffusion coefficient in the liquid can be estimated based on the Einstein–Stokes–Sutherland equation [51,52]

(A1) $$D=\frac{k_{b}T}{6\pi \eta r}$$

with the Boltzmann constant $k_{b}$, the temperature *T*, the dynamic viscosity of the melt η ($8.36\times 10^{-3}$ Pa s [53]) and the atomic radius *r* of the diffusing species (Al: $1.43\times 10^{-10}$ m). The temperature used is the liquidus temperature of CMSX-4^®^ (see Table 4)

## Appendix B. Derivation of the Advanced Scheil Model

The Scheil approach for the evolution of concentration in the solid and liquid during solidification is depicted in Figure A1.

First, the approach assumes that there is no diffusion in the solid and complete homogenization in the liquid. When solidification proceeds infinitesimally by $df_{s}$, the concentration in the liquid is raised by an amount of $dc_{l}$ by rejected elements from the solid. The amount of elements rejected must be equal to the amount arriving in the liquid. This means that the areas $A_{1}$ and $A_{2}$ must be equal. This leads to

(A2) $$\left(c_{l}-c_{s}\right)df_{s}=f_{l}dc_{l}$$

with the liquid fraction $f_{l}$. With the partition coefficient $k=c_{s}/c_{l}$ and $f_{s}+f_{l}=1$, this can be written as

(A3) $$c_{l}\left(1-k\right)df_{s}=\left(1-f_{s}\right)dc_{l}$$

Up to now, this was the classical approach. Normally the concentration in the liquid is not completely mixed. Thus, a concentration redistribution for the liquid can be done to account for this. For the sake of simplicity, a simple pile-up approach is used. The length of the liquid boundary layer where the concentration is increased in the liquid can be expressed by Equation (4). At the solid–liquid interface, the concentration is maximal. Then, it decreases linearly to $c_{0}$ at a distance of the equivalent boundary layer $\delta_{c}$. Essentially, the length of that boundary layer is defined by the ratio between the diffusion and solidification velocity. Finally, the area $A_{3}$ can be defined, where the concentration in the liquid is increased. For redistributing the elements from area $A_{2}$ to $A_{3}$, they are set as equal.

(A4) $$0.5dc_{l,D}f_{l,D}=\left(1-f_{s}\right)dc_{l}$$

Here, it is assumed that $f_{l,D}$ describes the part of the liquid fraction, where the concentration is increased, and $f_{l,R}$ is the part where no change in concentration occurs. Thus, $f_{l,D}+f_{l,R}+f_{s}=1$. Consequently, $dc_{l,D}$ can be expressed by

(A5) $$dc_{l,D}=\frac{2\left(1-f_{s}\right)dc_{l}}{f_{l,D}}$$

As for $c_{l}$, the boundary condition for $c_{l,D}$ is $c_{l,D}=c_{l}=c_{0}$ at $f_{s},x=0$. Therefore, $c_{l}$ is calculated as an intermediate parameter for calculating $c_{l,D}$, which is then used to define the concentration between dendrites. In the end, the concentration evolution in the solid is obtained by the classical approach ($c_{s}=kc_{l}$), whereas the concentration in the liquid is obtained by redistributing the elements in the liquid state to account for non-homogeneous concentration distributions.

## Appendix C. Simulation Overview

Figure A2 shows a snapshot of the simulation of the temperature field at the highest melt-pool depth. The point where the temporal evolution of the temperature at the melt pool bottom is tracked is also depicted.

Figure A3 and Figure A4 show the evolution over time when solidification starts with the liquidus position (measured as the distance to the maximum melt-pool depth), liquidus velocity (used as solidification velocity), cooling rate, and temperature gradient (measured at the current liquidus position). The solidification velocity was determined based on the cooling rate $\dot{T}$ and the absolute value of the temperature gradient |∇T| (see Equation (A6)).

(A6) $$v=\frac{\dot{T}}{\left|\nabla T\right|}$$

## Appendix D. Angular Dependency

A schematic representation of the calculation of the effective dendrite arm spacing $\lambda_{\alpha }$ for a given grain orientation (compare Equation (8)) based on the local temperature gradient is depicted in Figure A5.
